# Xenograft Zebrafish Models for the Development of Novel Anti-Hepatocellular Carcinoma Molecules

**DOI:** 10.3390/ph14080803

**Published:** 2021-08-16

**Authors:** Federica Tonon, Rossella Farra, Cristina Zennaro, Gabriele Pozzato, Nhung Truong, Salvatore Parisi, Flavio Rizzolio, Mario Grassi, Bruna Scaggiante, Fabrizio Zanconati, Deborah Bonazza, Gabriele Grassi, Barbara Dapas

**Affiliations:** 1 Department of Medical, Surgical and Health Sciences, University of Trieste, Cattinara Hospital, Strada di Fiume, 447, I 34149 Trieste, Italy; ftonon@units.it (F.T.); rfarra@units.it (R.F.); cristina.zennaro@asugi.sanita.fvg.it (C.Z.); lasignoradellago@hotmail.com (G.P.); fabrizio.zanconati@asugi.sanita.fvg.it (F.Z.); deborah.bonazza@asugi.sanita.fvg.it (D.B.); 2 Stem Cell Research and Application Laboratory, VNUHCM, University of Science, Ho Chi Minh City 72711, Vietnam; thnhung@hcmus.edu.vn; 3 Pathology Unit, CRO Aviano, National Cancer Institute, IRCCS, I 33081 Aviano, Italy; SALVATORE.PARISI@phd.units.it (S.P.); flavio.rizzolio@unive.it (F.R.); 4 Doctoral School in Molecular Biomedicine, University of Trieste, I 34127 Trieste, Italy; 5 Department of Molecular Sciences and Nanosystems, Ca’ Foscari University of Venice, I 30170 Mestre, Italy; 6 Department of Engineering and Architecture, University of Trieste, Via Valerio 6/A, I 34127 Trieste, Italy; MARIO.GRASSI@dia.units.it; 7 Department of Life Sciences, Cattinara University Hospital, Trieste University, Strada di Fiume 447, I 34149 Trieste, Italy; bscaggiante@units.it (B.S.); bdapas@units.it (B.D.)

**Keywords:** zebrafish, hepatocellular carcinoma, xenograft

## Abstract

Hepatocellular carcinoma (HCC) is the sixth most common type of tumor and the second leading cause of tumor-related death worldwide. Liver cirrhosis is the most important predisposing factor for HCC. Available therapeutic approaches are not very effective, especially for advanced HCC, which is the most common form of the disease at diagnosis. New therapeutic strategies are therefore urgently needed. The use of animal models represents a relevant tool for preclinical screening of new molecules/strategies against HCC. However, several issues, including animal husbandry, limit the use of current models (rodent/pig). One animal model that has attracted the attention of the scientific community in the last 15 years is the zebrafish. This freshwater fish has several attractive features, such as short reproductive time, limited space and cost requirements for husbandry, body transparency and the fact that embryos do not show immune response to transplanted cells. To date, two different types of zebrafish models for HCC have been developed: the transgenic zebrafish and the zebrafish xenograft models. Since transgenic zebrafish models for HCC have been described elsewhere, in this review, we focus on the description of zebrafish xenograft models that have been used in the last five years to test new molecules/strategies against HCC.

## 1. Introduction

Despite the considerable progress made in recent years in understanding the risk factors, epidemiology and molecular features of hepatocellular carcinoma (HCC), this pathology remains a leading cause of cancer-related deaths in several parts of the world [1]. It is therefore evident that further improvement in the understanding of the disease and identification of new therapeutic molecules/strategies are urgently needed. In this regard, the use of animal models represents a relevant tool. Rodents are most commonly used for preclinical screening of drugs. However, the husbandry of these animals is not free of limitations, and rodent models do not necessarily reflect the pathophysiology of human cancers. In contrast, pigs represent a more predictive model for drug selection than rodents [2]. Unfortunately, the cost and husbandry requirements of this animal model make it unsuitable for large-scale drug screening. An animal model with several attractive features has emerged in the last 15 years [3] and has attracted the interest of the scientific community: the zebrafish. In this review, we focus on the papers published in the last five years that use the zebrafish to develop new strategies against HCC; the advantages and disadvantages of the described approaches are also highlighted. While transgenic tumor models of HCC have already been reported [4], here we focus on the description of the zebrafish xenograft model of HCC. Before presenting the experimental work performed, some details about HCC and the general characteristics of the zebrafish model are described.

### 1.1. Hepatocellular Carcinoma

HCC is the sixth most common type of tumor and the second leading cause of tumor-related death worldwide (https://gco.iarc.fr/today/data/factsheets/populations/900-world-fact-sheets.pdf, accessed on 14 July 2021). The prognosis is very poor with a 5-year overall survival rate of less than 12% [5]. The incidence of the disease reflects the frequency of HCC risk factors [6]. Alcoholic liver disease (ALD), which can progress to cirrhosis; hepatitis C virus (HCV) infection; and nonalcoholic fatty liver disease (NAFLD) are common risk factors in Europe, North America, and Japan. ALD and HCV infection, either alone or in combination, account for more than two-thirds of all HCC cases in the Western world [7], where, however, HCC is less common than in Eastern countries [8]. Exposure to aflatoxin B1 together with chronic hepatitis B virus (HBV) or HCV infections are relevant risk factors for the development of HCC in Asia and at Sub-Saharan Africa [9,10]. Liver cirrhosis, which can result from any of the above risk factors, is the most relevant predisposing element for HCC [11]. In addition, human immunodeficiency virus (HIV) is recognized as a common cofactor that increases the risk of HCC in patients with chronic hepatitis B virus (HBV) or HCV infection [12]. Finally, tobacco use, autoimmune hepatitis, alpha-1 antitrypsin deficiency, and hereditary hemochromatosis [9] are other risk factors that promote HCC.

HCC cells have abnormal cell-cycle control and evade apoptosis [13]. In addition, this tumor type is characterized by aberrant and exuberant neo-angiogenesis represented mainly by arterial-like vessels; in contrast, normal liver is essentially supplied by venous vessels [14]. The formation of tumor-derived arterial vessels increases with disease progression and predominates in advanced HCC [15]. This is related to the increased oxygen demand of the large lesions. Another difference between HCC and normal liver vasculature is the increased leakage [16] of HCC vasculature. This is related to abnormalities in the endothelial layer, but also to the frequent absence of pericytes and/or vascular smooth muscle cells around the endothelial layer.

### 1.2. Available Treatments

The lethality of HCC is mainly related to the fact that the disease is often diagnosed at a late stage, when surgical resection of the tumor is no longer possible. Surgical resection, transplantation, and ablation are treatments that have a high rate of complete clinical response with proven curative potential and are generally limited to well-defined stages of HCC [17,18]. In patients with early stage HCC without cirrhosis, tumor resection is the treatment of choice; however, this clinical situation occurs in less than 5% of cases [9]. The possibility of performing liver transplantation in HCC patients with or without cirrhosis has completely changed the treatment strategy. However, the lack of donors usually leads to a time delay, which allows the progression of HCC and makes transplantation difficult [19]. Another potential therapeutic approach is local radiofrequency ablation (RFA). RFA can be performed percutaneously under ultrasound or computed tomography guidance [20]. RFA causes thermal necrosis of the tumor by delivering electromagnetic energy through single or multiple needle electrodes [21].

The only non-curative treatments that can improve survival and maintain a good quality of life in patients with intermediate to advanced-stage HCC are transarterial chemoembolization (TACE) and chemotherapy [22,23]. TACE technique, the standard clinical treatment for intermediate-stage HCC patients, involves the combination of selective injection of antineoplastic agents through the hepatic artery and selective obstruction of tumor supply vessels. The combination of TACE with the multi-kinase inhibitor sorafenib has a strong rationale based on the effect that chemoembolization exerts on angiogenesis in the tumor; however, this procedure is associated with a high rate of adverse events [9].

Systemic chemotherapy may be considered for un-resectable HCC. However, it should be noted that the disease has a high resistance to chemotherapy and radiation [20,24]. Systemic chemotherapy with sorafenib has some beneficial effects. Sorafenib is an oral multi-kinase inhibitor that blocks the Raf/MEK/ERK pathway and other extracellular receptor tyrosine kinases. Its action results in anti-proliferative and anti-angiogenic activity that can delay tumor progression [13]. Sorafenib is the standard systemic therapy for patients with advanced HCC and well-preserved liver function, as well as for patients with intermediate-stage HCC who progress after TACE [25]. It should be noted that the clinical benefit of sorafenib is modest, as it prolongs both relapse-free survival and overall survival by only 2 to 3 months, with a response rate of less than 5% [24,26]. Moreover, the incidence of adverse toxic events (gastrointestinal and dermatological) after treatment is high (~80%).

Recently, four new chemotherapy molecules have become available for clinical use [27]: lenvatinib, as a first-line agent; and regorafenib, ramucirumab and cabozantinib, as second-line agents. Lenvatinib is an oral kinase inhibitor that selectively inhibits receptor tyrosine kinases involved in angiogenesis and malignant transformation of tumors. Regorafenib is an oral inhibitor of multiple protein kinases, including VEGFR1, VEGFR2, VEGFR3, TIE2, PDGFRβ, FGFR, KIT, RET, RAF-1 and B-RAF. Ramucirumab is a recombinant human immunoglobulin IgG1 monoclonal antibody that inhibits VEGFR-2. Finally, cabozantinib is an oral multikinase inhibitor of VEGF, c- MET, RET, TIE2, FLT3 and of the TAM family of receptor kinases. The efficacy of these new drugs needs to be defined in the real world.

### 1.3. Zebrafish

The zebrafish (*Danio rerio*) is a small freshwater fish belonging to the family of Cyprinidae; it is found in the wild in the rivers of Northern India, Bangladesh and parts of Southern Nepal [28]. The zebrafish is a diurnal predator of aquatic invertebrates that reproduces by externally fertilized eggs.

**Figure 1:**
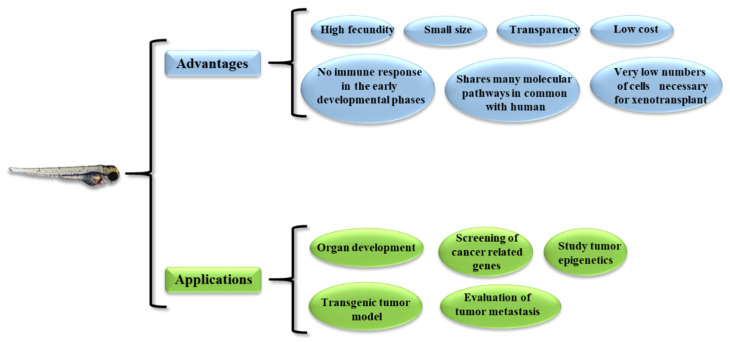
Advantages and possible application of the zebrafish model.

In 1981 [29], the idea to use the zebrafish as a novel vertebrate model organism to study human diseases and test innovative therapeutic molecules/strategies was first proposed. There are several reasons for its use in biomedical research [30,31] (Figure 1). First, zebrafish has a high fecundity, so many animals can be produced in a short time; moreover, both embryos and adults have a small size, so little space is needed for housing. Second, the transparency of both embryos and larvae allows the fate of the injected tumor cells (growth and migration) to be tracked within the animal body. In particular, a genetic strain has been developed that retains much of its transparency into adulthood; however, adults are less transparent than larvae [32]. Third, zebrafish lack an adaptive immune response until about 4 weeks after fertilization, so xenotransplanted cells are not rejected at this time [33]; an immunosuppressed transgenic mutant zebrafish is available if older animals are needed [34]. Finally, the cost of maintaining the zebrafish is much more manageable compared to other animal models, such as rodents.

Other characteristics make the zebrafish an attractive animal model for biomedical studies. For example, the zebrafish shares molecular signaling pathways with humans, so, although it is a fish, it bears a significant similarity to humans. In this regard, it should be noted that the zebrafish shares a great similarity with the human liver [35], as it contains the same cell types found in the human liver (hepatocytes, stellate cells, biliary cells and endothelial cells). In addition, a Tg(fli:EGFP) zebrafish strain was generated whose endothelial cells constitutively express Enhanced Green Fluorescence Protein (EGFP) [36]. This can be used, for example, to study the effects of anti-angiogenic drugs, as well as tumor neo-angiogenesis [37]. While zebrafish larvae develop best at 28 °C, it has been observed that it is possible to maintain zebrafish at 32.5–35 °C, allowing both animal and human xenograft cells to survive [38]. Finally, a very small number of cells is required to enable the generation of the xenograft (about a few hundred cells). All of these features make zebrafish models particularly suitable for high-throughput drug screening.

From a practical perspective, zebrafish models can be used to (Figure 1) (1) study the development of organs similar to their mammalian counterparts, (2) generate transgenic tumor and xenograft models, (3) study the metastatic behavior of tumors, (4) screen cancer-related genes, and (5) study epigenetic mechanisms.

Since the first demonstration of the possibility to generate a xenograft in zebrafish by using human cells [3], the field has developed very rapidly. Nowadays, different cell injection sites, such as yolk sac, pericardium, intra-peritoneal cavity, subcutaneous and sub-intestinal veins, have been performed [31]. Interestingly, improvements were also made regarding the origin of the cells to be xenotransplanted. Initially, human cancer cell lines were used, leading to the so-called cell-line-derived xenograft (CDX) models [30]. Human cancer cell lines are easy to obtain and are very suitable for replication experiments. However, continuous in vitro passage may alter their phenotypic/genetic pattern, moving them away from the original tumor characteristics. To circumvent this aspect, patient-derived tumor cells have been used to generate xenograft zebrafish models (Patient-Derived Tumor Xenograft (PDX) model) [30]. A major advantage of PDX is that only a few patient-derived cells are needed to generate PDX in zebrafish, as compared to other animal models, such as rodents, which are often difficult to use for this purpose.

## 2. Xenograft Zebrafish Models of HCC

As reported above, we here focus on papers describing works with the zebrafish xenograft model of HCC, together with one noticeable example of zebrafish liver fibrosis (LF), the most common condition predisposing to HCC development. For a more rational approach to the topic, we have divided the papers into three categories: the first, where the main objective was to investigate the effects of therapeutic molecules on the growth of implanted HCC tumor cells; the second, where drug effects on HCC cell dissemination was evaluated; and the third, where an anti-LF approach has been evaluated (see Table 1 for a synopsis of the papers presented). In addition, a synopsis of the different HCC cell lines used with their degree of differentiation is presented in Table 2.

### 2.1. Evaluation of Tumor Mass Growth

Liu et al. [39] studied the molecular mechanisms responsible for the anti-HCC properties of aloperine (ALO), a quinolizidine alkaloid from Sophora alopecuroides L, used in traditional Chinese medicine. In vitro, the authors used the HCC cell line HuH7 and Hep3B, which show an intermediate hepatic differentiation grade or are well-differentiated epithelial phenotype, respectively [52,53]. ALO inhibited cell growth by inducing G2/M cell cycle arrest via the expression downregulation of cdc25C, cdc2 and cyclin B1. ALO also induced apoptosis related to the loss of mitochondrial potential, the release of cytochrome c into cytosol and the increase of caspase-9, caspase-3 and PARP cleavage. For the in vivo experiments, two-days-post-fertilization (dpf) embryos were micro-injected into the yolk sac with CM-Dil-labeled Huh7 (200 cells per embryo). Embryos were exposed to increasing concentrations of ALO by soaking. Compared to control, at 5 dpf, it was possible to detect a dose-dependent reduction in the HuH7 tumor mass, as evaluated by fluorescence microscopy. Unfortunately, only representative imagines were reported with no numerical quantification of the tumor-mass reduction. Thus, it is somewhat difficult to compare the extent of the in vitro inhibition with that in vivo. Additionally, no quantification (mRNA levels) of the target downregulated in vitro were reported, again making the comparison with the in vitro data not easy. Despite these aspects, the data suggest that ALO has the potential to downregulate the growth of HCC cells in vivo.

Chang et al. [40] evaluated the anti-HCC properties of 4-phenoxyphenol derivative, 4-[4-(4-hydroxyphenoxy)phenoxy]phenol (4-HPPP). Moreover, 4-HPPPs belong to phenolic compounds, which can be found in many plants; they have been reported to modulate key proteins regulating differentiation, proliferations, metastasis and apoptosis for the treatment in cancer [60]. In vitro, the authors show that 4-HPPP exerts a significant inhibitory effect on the proliferation of HuH7 also promoting apoptosis via DNA damage pathway. For the test in zebrafish, HuH7 cells (200/embryo) were labeled with DiI and injected into the yolk sac of 2-dpf-old embryos, which were then exposed to 1 μM of 4-HPPP for one or two days. Despite that a tendency towards the reduction in HuH7 mass was observed, no statistical significance was reached, as compared to the control treated embryos. In this regard, it should be noted that, in cultured HuH7, the maximal anti-proliferative activity was observed by using 5–10 μM 4-HPPP (60–80% decrease); with 1 μM 4-HPPP, the decrease was of only 20%, i.e., not dissimilar to that observed in zebrafish. Thus, it may be possible that suboptimal conditions were undertaken for the zebrafish evaluation.

Another polyphenolic compound tested for its anti-HCC properties is propyl gallate (PG). This is an antioxidant synthesized by the condensation of gallic acid and propanol, commonly used in the preservation of food and medicinal preparations. Although it has anticancer activities [61], its role in HCC is largely unknown. Wei et al. [41] demonstrated that PG effectively inhibited the proliferation of cultured Hep3B and HepJ5 in a dose- and time-dependent manner. Notably, the effect was more evident in the poor differentiated HepJ5 (invasive HCC cells [54]), compared to the well-differentiated Hep3B. The downregulation of cell growth was mainly due to the enhancement of ROS production and autophagy activation. At 2 dpf, zebrafish embryos were injected with approximately 200 cells (either Hep3B or HepJ5 stained by the CFSE die) in the yolk sac. The embryos were then treated by dH_2_O (control) or PG at doses ranging from 10 to 40 μg/mL. Fluorescent cells of embryos were checked at 2 h post-implantation and at 1–3 days post-injection (dpi) by fluorescence microscopy. At the dose of 10 μg/mL PG, we confirmed the increased sensitivity of HepJ5 vs. Hep3B, while, at 40 μg/mL PG, there were no substantial differences, suggesting that, at a high dose, PG may be effective in both differentiated and less differentiated forms of HCC.

A molecule similar to PG, but differing for the link with methyl groups, is Methyl gallate (MG). Derived from plant phenolic gallic acid, MG is generally recognized as safe, with antioxidant [62] and antitumor properties [63]. Huang et al. [42] showed that MG treatment inhibited the proliferation of the HCC cell lines Hep3B and HepJ5 cells in vitro. Despite its antioxidant properties, the authors surprisingly show that MG caused the increase of superoxide and oxidative stress, eventually resulting in autophagy induction. In 2-dpf zebrafish embryos, HepJ5 or Hep3B (200 cells/embryo) stained by CM-Dil were injected in the yolk. Subsequently, either distilled H_2_O or MG (40μg/mL) were administered to the embryos. HCC cells were observed at 2 h post-implantation, and then 1 and 3 days post-injection (dpi), by fluorescence microscopy. The authors could confirm the effectiveness observed in vitro, thus demonstrating a good correlation vitro/vivo and confirming the anti-HCC property of MG.

Theabrownin (TB), the main pigment and bioactive component of tea, can be synthesized from the oxidation and polymerization of tea polyphenols [64]. Its anticancer potential has been proposed [65]; however, the effectiveness in HCC needs investigation. In cultured HCC cell lines, HepG2 (well differentiated HCC cells [55]), HuH7 and SKHep1 (cell line of endothelial origin, not of HCC origin [56]), Xu et al. [43] showed that TB (200 μg/μL) potently reduced cell viability, as evaluated by MTT test. This was due to the activation of the JNK signaling pathway that resulted in an anti-proliferative and pro-apoptotic effect. In vivo, the authors microinjected into the yolk sac of embryos (2 dpf), HuH7 cells (200 cells/fish) stained by CM-Dil. One day after (3 dpf), tumor mass size was measured (by fluorescence microscopy) and subsequently larvae were treated by TB (16.7 μg/mL). TB significantly inhibited HuH7 growth with an inhibitory rate of 48.1%, which resulted to be more pronounced than that measured in vitro by MTT at the same TB dose. Whereas the reasons for this discrepancy remain unclear, it cannot be excluded that the culture medium might have in part inactivated TB.

**Figure 2:**
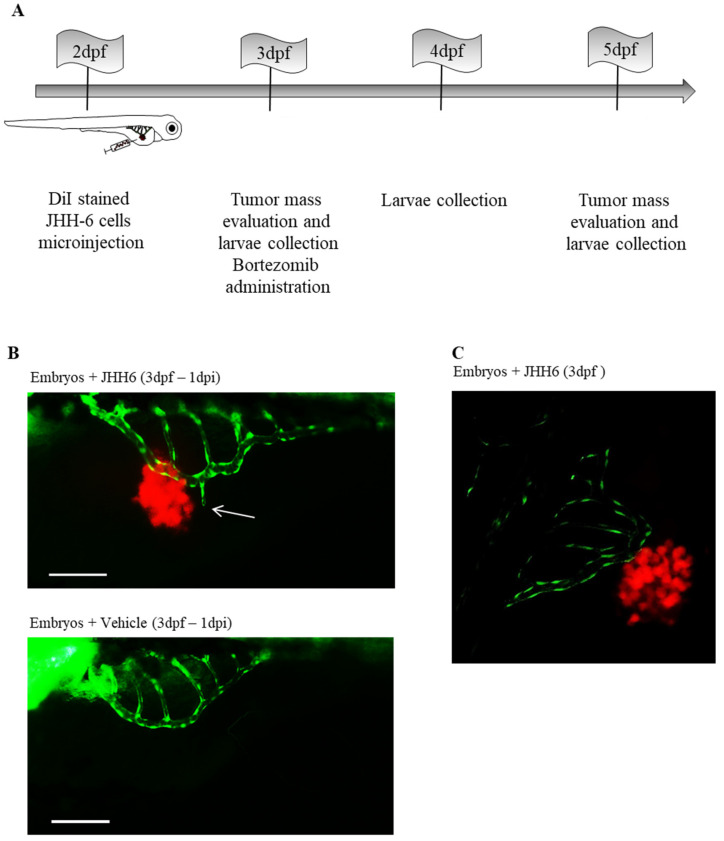
Neo-angiogenesis in zebrafish embryos microinjected with JHH6 cells. (**A**) Schematic representation of the protocol used to create the xenograft model of HCC in zebrafish embryos. DiI-stained JHH6 cells were microinjected into 2-dpf-old embryos’ yolk. Animals were then maintained at 34 °C until the end of the experimental protocol (5 dpf). At 1 and 3 days post-tumor-cell-microinjection (1–3 dpi), images of the tumor masses were acquired by fluorescent microscope (Leica DM2000), and larvae were also collected to be analyzed. (**B**) Zebrafish neo-angiogenesis in larvae injected with JHH6 cells was examined by using the transgenic zebrafish strain Tg(fli1:EGFP). A representative zebrafish larva injected with stained JHH6 cells (red) is shown above. The image shows the vessels of the SIVP (green) near to the cancer cells (the white arrow shows a new branch of the SIVP); as control, a larva injected with vehicle alone is reported (bottom). Bar = 200 μm, dpf = day(s) after fertilization; dpi = day(s) after injection, SIVP = Sub-Intestinal Vessels Plexus. (**C**) Representative JHH6 mass (red) and neo-vessels (green) imaged with a confocal microscope; bar = 200 μm, dpf = day(s) post-fertilization; dpi = day(s) post-injection. (**B**,**C**) reproduced with permission from Reference [44].

We developed an HCC zebrafish xenograft model based on the use of JHH6 [44]. This is an HCC cell line with a low cellular differentiation level [57,58] that cannot be transplanted into mice (our personal observation). Thus, the zebrafish model we developed provides a valuable opportunity to test an HCC cell line in vivo that would otherwise only be tested in vitro; however, it is a relevant model for poorly differentiated HCC. The 2-dpf-old zebrafish embryos were microinjected in the yolk sac with JHH6 (500 cells/embryo) and stained with 2 μg/mL DiI. Tumor mass growth was evaluated in the three days following cells’ microinjection (Figure 2A).

Compared to 3 dpf, tumor mass increased by approximately 35% in 5 dpf, which was also confirmed by the increase in expression of the human proliferation marker Ki67. We also observed that the injected cells migrated into the tail of the larvae, suggesting a metastatic process. Using the fish strain Tg(fli1:EGFP)(y1), which expresses EGFP throughout the vasculature under the control of the fli1 promoter, we examined the effects of JHH6 on tumor neo-angiogenesis. We detected increased neo-vascularization toward JHH6 and also demonstrated the physical proximity of tumor neo-vessels to JHH6 by confocal microscopy (Figure 2B,C). Compared to the control, JHH6-injected zebrafish larvae exhibited thicker and more branched vessels that were also approximately one-third longer and one-quarter larger in diameter. In support of the above observations, we showed the increased expression of zebrafish VEGF-A, as well as its receptors VEGFR-1 and VEGFR-2. Taken together, these data suggest that our xenograft zebrafish model of HCC can recapitulate the potent pro-angiogenic power of HCC.

We then tested the anti-HCC potential of the drug bortezomib. This is a proteasome inhibitor that we have shown to possess anti-HCC properties in various HCC cell lines, including JHH6 in vitro [66,67,68]. At the dose used (20 nM), bortezomib did not induce significant signs of suffering (no pericardial edema) in larvae. Administration of bortezomib (3 dpf) for two days (5 dpf to 3 dpi) reduced the growth of JHH6 by approximately 50% compared to untreated larvae and also reduced the expression level of the human proliferation marker Ki67. Taken together, our results suggest that the zebrafish model of HCC we developed, based on the use of the aggressive HCC cell line JHH6, can be profitably used to simultaneously investigate the effects of conventional as well as experimental drugs on HCC cell growth and tumor neo-angiogenesis [69,70,71,72,73,74,75].

Recently [45], a zebrafish carrying a loss-of-function point mutation in the acetylcholinesterase (ACHE) gene was used to generate a xenograft zebrafish model of HCC. ACHE is responsible for the enzymatic degradation of acetylcholine (ACh), and a decrease in its activity is positively associated with HCC tumor size and stage [76]. The genetically modified zebrafish strain (achesb55−/−) has a null phenotype with respect to ACHE function due to the point mutation. At 2 dpf, 300 Hep3B or SKHep1 cells stained with DiI were injected into the yolk sac of each embryo. Hep3B and SKHep1 express ACHE at low and high levels, respectively; moreover, Hep3B is a well-differentiated epithelial HCC cell line [53], while SKHep1 is highly tumorigenic [77]. A comparison was made between homozygous (achesb55−/−) and heterozygous (achesb55+/−) with respect to tumor growth. Moreover, achesb55-/- embryos developed larger tumors than achesb55+/−, regardless of the injected cell line. This suggests that zebrafish expression ACHE rather than tumor production of ACHE influences tumor growth. Interestingly, the authors show that, regardless of the injected HCC cell type, metastasis was more frequent in achesb55+/− compared to achesb55−/− embryos. This may suggest that ACh promotes HCC cell growth but impairs cell migration. Alternatively, the authors suggest that it is possible that, in achesb55−/−, the lack of ACHE production causes cardiac edema and decreased blood flow, which promotes cell migration. If this proves true, this model may not be optimal for studying metastasis. Nevertheless, the model developed is interesting for testing drugs against HCC.

In a very articulated and interesting work, Lin et al. [46] tested the anti-HCC potential of sorafenib in comparison with two other multi-tyrosine kinase inhibitors, namely 419S1 and 420S1. The peculiarity of the work is that the authors used, among others, cells from 15 different HCC patients that generated a so-called PDX. Xenotransplantation was performed in 2-dpf embryos, with 200 cells injected into the yolk sac for each different patient; 1-dpi larvae were treated with the drugs for two days (3 dpi). The authors found that the efficacy of 419S1 and 420S1 in preventing liver cancer proliferation was superior to that of sorafenib; however, they also found differential efficacy in the cells of the different patients. In addition, they showed that both 419S1 and 420S1 can downregulate HCC cell migration. Despite some limitations with PDX (see Section 3—Conclusions), it is clear that the ability to test HCC cells derived from a defined patient opens the way to personalized medicine.

### 2.2. Evaluation of Tumor Cell Migration

Yang et al. [47] focused their attention on the downregulation of HCC metastasis. For this purpose, they tested honokiol, a component isolated from the root and stem bark of magnolia. Honokiol has anti-thrombocytic, antibacterial, anti-inflammatory and antitumor properties [78]. Due to its poor water solubility, honokiol was delivered to red fluorescently labeled HepG2 cells (HepG2-Red) via PEG modified liposomes; an empty liposome was used as a control. LH (liposomal honokiol) was administered at a concentration of 40 μM, since, at this concentration, the anti-migration effect on HepG2 was maximal in vitro, without inducing cell death. Indeed, the authors’ aim was to find an anti-metastasis approach with the least toxic effects. After six hours of treatment by LH, HepG2-Red cells were injected into the bloodstream (50–100 cells per embryo) of Tg (kdr1: EGFP) zebrafish embryo. Thus, it was possible to follow both HCC-Red cells and vascular green cells. Forty-eighth hours after injection, 40% of untreated HepG2-Red cells extravasated from host vessels and migrated to adjacent tissues. In contrast, only 5% of the pretreated (by LH) HepG2-Red cells extravasated. The authors demonstrated that the reduction in migration was due to inhibition of Rac1 and Cdc42 activity by LH; this is consistent with the knowledge that Rac 1 and Cdc42 are important regulators of the actin cytoskeleton in various cellular functions, including cell migration. In addition, LH was observed to target the EGF-EGFR pathway and affect the downstream signaling pathway of PI3K/Akt, ERK and JNK. Finally, LH inhibited the expression of matrix metalloproteinase-2 and -9, two of the major metastasis-promoting molecules that degrade the extracellular matrix and promote cell metastasis. The approach taken by the authors is particularly interesting, as it aims to downregulate the metastatic process, thus minimizing side effects. These beneficial properties can be further enhanced by delivering the therapeutic molecules via HCC-targeted delivery systems [79].

Thioredoxin-interacting protein (TXNIP) is a redox-sensitive transcription factor that promotes the increase of ROS and activation of apoptotic response [80]. Increased TXNIP expression in HCC cell lines, such as HuH7, HepG2 and Hep3B, is correlated with proliferation inhibition [81]. Its role in metastasis in HCC was investigated by Gunes et al. [48]. In vitro, the authors showed that TXNIP overexpression promotes migration and invasion of HepG2 and HuH7. Moreover, TXNIP overexpression was shown to induce sprouting and tubule formation and promote branched tubulogenesis in HuH7 and HepG2 cells. Accordingly, inhibition of TXNIP expression resulted in the downregulation of cellular motility and invasion. To verify these observations in vivo, HepG2 cells overexpressing TXNIP and labeled with DiO were injected into zebrafish embryos. Approximately 200–300 HCC cells were injected into the central portion of the yolk sac of 2-dpf-old zebrafish embryos. After 3 dpi, control HepG2 cells metastasized to the tail/head of the embryo in 18% of xenografts, whereas TXNIP-transfected HepG2 cells metastasized to 30% of xenografts. These data suggest that TXNIP plays a role as a metastasis promoter in HCC, so anti-TXNIP molecules may be of potential therapeutic value for HCC.

Moreover, p73 is closely related to the tumor suppressor gene p53 [82]. Thus, p73 has been originally proposed to have tumor suppressor properties. However, its role in tumors in general and in HCC in particular is not clear [83]. The role of TAp73 isoforms in HCC was recently investigated by Iscan et al. [49]. The authors showed that the TAp73 β isoform is strongly induced in HCC and that it is associated with poor patient survival. In vitro assays showed that overexpression of the TAp73β isoform in HCC cells downregulated the expression of cell-cycle-regulatory genes, such as those involved in G1/S and G2/M checkpoints; this resulted in an increase in G0/G1 phase cells at the expense of S and G2/M phase cells. Interestingly, no cell death was induced. Another interesting finding was that overexpression of TAp73 β induced de-differentiation of HCC cells via repression of hepatocyte lineage markers. The HCC cell line Hep3B expressing TAp73 β via an inducible promoter (regulated by doxycycline) was stained with DiI and injected into the yolk sac of 2-dpf-old zebrafish embryos (approximately 300 cells/embryo). The next day, one group of larvae was treated with doxycycline for three days, while another group was not treated. A cell migration of 44% was observed in doxycycline-treated larvae (overexpression of TAp73 β), while the value in non-treated larvae (no overexpression of TAp73 β) was 21%. These observations support the relevant contribution of TAp73 β to HCC cell migration in the zebrafish xenograft model. Together, the results obtained suggest that, in HCC, the oncogenic effects (de-differentiation and pro-migratory effects) of TAp73β overcome the tumor-suppressor effect (anti-proliferative effect), possibly explaining the worse patient prognosis.

The HOXC cluster [84] encodes the HOX transcript antisense intergenic RNA (HOTAIR). HOTAIR is a 2148-nt-long spliced and poly-adenylated long non-coding RNA (lncRNA) whose overexpression has been associated with poor prognosis, invasiveness and aggressiveness in cancer [85]. Topel et al. [50] investigated the role of HOTAIR in HCC. In vitro, the authors showed that HOTAIR promotes the expression of both epithelial and mesenchymal markers in HCC cells. This is very interesting because tumor cells that simultaneously express mesenchymal and epithelial markers contribute significantly to metastasis [86]. In addition, the authors show that HOTAIR impairs cell adhesion. These phenotypes were induced by downregulation of c-Met by HOTAIR. Moreover, c-Met is a receptor tyrosine kinase known to be upregulated in liver disease, promotes hepatocyte proliferation and is responsible for the initiation and development of HCC [87]. To prove the above observations in vivo, the authors used the HCC cell line SNU-449 (intermediate/low differentiation level [59]) and the same cell line overexpressing HOTAIR (SNU-499/HOTAIR) to generate a zebrafish xenograft. At 2 dpf, SNU-449 or SNU-499/HOTAIR stained with DiI was injected into the yolk sac of the embryos (100 cells/embryo). Metastatic capacity was calculated based on the number of xenografts that metastasized after 4 dpi of cells. SNU-499/HOTAIR showed almost twice the metastatic potential compared to SNU-499, demonstrating the relevance of HOTAIR to the metastatic power of the HCC cell line SNU-449. This observation opens the way for the development of novel anti-metastatic drugs in HCC, whose potential toxic effect needs to be carefully evaluated, as physiological cell migration is relevant in many normal tissues.

### 2.3. Evaluation of an Anti-Liver Fibrosis Approach

Chronic hepatitis B/C virus infection, alcohol abuse and nonalcoholic fatty liver disease are major causes of liver fibrosis (LF), which inevitably leads to progressive impairment of liver function (cirrhosis), often culminating in HCC. LF is a major public health problem worldwide, with more than 800 million people affected and a mortality rate of approximately 2 million deaths per year [88]. Therefore, treatment of LF may help to reduce progression to HCC. In this context, Van der Helm et al. [51] developed a novel zebrafish model of liver fibrosis. Although available, mouse models of liver fibrosis are far less suitable for screening purposes than a zebrafish model. It is noteworthy that the zebrafish bears a good resemblance to the human liver [35]. To generate the zebrafish model of liver fibrosis, the authors tested carbon tetrachloride (CCL4), which is commonly used to induce fibrosis in mouse liver, and thioacetamide (TAA). While CCL failed to induce liver fibrosis in zebrafish, TAA did. To this end, 2-dpf zebrafish embryos were treated by adding TAA to water for 6 days. Histological analysis revealed the presence of collagen fibers between hepatocytes, in parallel with increased expression of collagen mRNA. Moreover, the expression of the pro-fibrotic cytokine TGF-β was increased in the group treated with TAA, compared to the control. Since previous studies [89,90] indicated that both mouse and human mesenchymal stromal cells (MSCs) can reduce fibrosis in mouse models of liver fibrosis, the authors tested MSCs as a potential strategy to downregulate TAA-induced liver fibrosis. MSCs from mice (labeled with red fluorescent protein—RFP), fibroblasts or a solvent control were injected (100 cells/embryo) in close proximity to the liver 3 days after treatment with TAA (5 dpf). MSCs reduced RNA-expression levels of collagen and TGF-β compared to embryos without MSC treatment; however, fibroblasts or a solvent control resulted in intermediate reductions in these genes. These observations raise concerns about the specificity of the effect of MSCs. Moreover, MSCs and fibroblasts were able to convert the pathological liver structure into a more normal liver tissue architecture and also reduce collagen deposition. The authors propose that the beneficial effect of MSCs may be due to the expression of various factors (such as HGF, IGF-1 and VEGF) involved in tissue regeneration and reversal of fibrosis. Despite some uncertainties regarding the specificity of the effect of MSCs, it is evident that the developed zebrafish model may be of practical use to test anti-LF strategies potentially leading to the prevention of HCC development.

## 3. Conclusions

HCC is one of the most common causes of cancer-related death in different parts of the world [1]. Therefore, additional efforts to identify new therapeutic options are urgently needed. Although several animal models exist to study HCC, these models are often not suitable for large-scale drug screening due to various problems, including animal husbandry. One animal model has attracted the attention of the scientific community over the past 15 years [3]: the zebrafish model. This animal model has many interesting features, such as a short reproduction time, low space and cost requirements for husbandry, body transparency that allows tracking of fluorescently labeled xenotransplanted cells, and the fact that embryos do not show an immune response to transplanted cells. Limitations include the following: (1) the microenvironment in which the xenotransplanted HCC cells develop was not well characterized, (2) the zebrafish vessels may not fully recapitulate the arterial-like supply of HCC, (3) quantification of the proteins of interest by conventional Western blotting may be extremely difficult due to the limited number of injected cells, and (4) there may be technical problems in amplifying the target human gene due to the potentially compromising effect of the large excess of zebrafish mRNA. Some of the above limitations may be overcome, but other, probably not so easily. The characterization of the microenvironment where xenotransplanted HCC cells develop can be achieved by biochemical/microscopy/immunohistochemistry analysis. For protein quantification in the transplanted cells, techniques alternative to Western blotting, such as immunohistochemistry, may be tested. The use of in situ hybridization techniques (labeled antisense nucleic acid molecules) to identify the target mRNA present in the xenotransplanted cells may be useful to overcome the presence of the large excess of zebrafish mRNA. An alternative may be represented by digital droplex PCR, which usually shows increased sensibility compared to the classical quantitative real time PCR. In contrast to the above comment, it is difficult to imagine an easy solution to the fact that zebrafish vessels may not fully recapitulate the arterial-like supply of HCC. Thus, this limitation should be kept in mind when drawing conclusion about any anti-angiogenic approach for HCC obtained in the zebrafish model. Finally, the choice of HCC cell line may also have a relevant impact on the results, as cells with different phenotypes may behave differently. Therefore, it would be desirable to test the xenograft model with at least two HCC cell lines with significantly different phenotypes.

Despite the above considerations, we believe that, by considering the model limitations, it will be possible to adequately use the zebrafish model for preclinical screening of novel anti-HCC molecules/strategies.

## Figures and Tables

**Table 1:** HCC Xenograf zebrafish models.

Author	Agent Investigated	Effects In Vitro	Cell Line Xenografted	Number of Cells	Injection Site	Effects In Vivo
Liu et al [39]	Aloperine	G2/M cell block, apoptosis	HuH7	200	Yolk sac	Reduced tumor growth
Chang et al [40]	4-HPP	Inhibition of proliferation, apoptosis	HuH7	200	Yolk sac	Tendency to tumor growth reduction
Wei et al [41]	Propyl gallate	Inhibition of proliferation, authophagy	Hep3B HepJ5	200	Yolk sac	Reduced tumor growth
Huang et al [42]	Methyl gallate	Inhibition of proliferation, authophagy	Hep3B HepJ5	200	Yolk sac	Reduced tumor growth
Xu et al [43]	Theabrownin	Inhibition of proliferation, apoptosis	HuH7	200	Yolk sac	Reduced tumor growth
Tonon et al [44]	Bortezomib	Inhibition of proliferation, apoptosis	JHH6	500	Yolk sac	Reduced tumor growth, anti-angiogenic
Avci et al [45]	Acetylcholine		Hep3B SKHep1	300	Yolk sac	Increased tumor growth, reduced metastasis
Lin et al [46]	419S1, 420S1 multikinase inhibitors		Patient-derived HCC cells	200	Yolk sac	Reduced tumor growth and metastasis
Yang et al [47]	Honokiol	Anti-migratory effects	HepG2	50-100	Blood circulation	Reduced tumor metastasis
Gunes et al [48]	Thioredoxin-interacting protein	Promotion of migration	HepG2	200-300	Yolk sac	Increased tumor metastasis
Iscan et al [49]	Tap73 isoform	G0/G1 cycle arrest, de-differentiation	Hep3B	300	Yolk sac	Increased tumor metastasis
Topel et al [50]	HOTAIR lncRNA	Expression of epithelial/mesenchymal markers, impaired adhesion	SNU-499	100	Yolk sac	Increased tumor metastasis
Van der Helm et al [51]	Mesenchymal stromal cells	Expression of HGF, IGF-1, VEGF		100	Close proximity to the liver	Reduction of liver fibrosis

**Table 2:** HCC cell lines employed to generate HCC xenograft zebrafish model.

Cell Line Xenografted	Differentiation Grade	Reference
HuH7	Intermediate	[52]
Hep3B	High	[53]
HepJ5	Low	[54]
HepG2	High	[55]
SKHep1	Endothelial origin	[56]
JHH6	Low	[57,58]
SNU-499	Intermediate Low	[59]

## Data Availability

Data sharing not applicable.

## References

[1] Yang J.D., Hainaut P., Gores G.J., Amadou A., Plymoth A., Roberts L.R. (2019). A global view of hepatocellular carcinoma: Trends, risk, prevention and management. Nat. Rev. Gastroenterol. Hepatol..

[2] Segatto N.V., Remiao M.H., Schachtschneider K.M., Seixas F.K., Schook L.B., Collares T. (2017). The Oncopig Cancer Model as a Complementary Tool for Phenotypic Drug Discovery. Front. Pharmacol..

[3] Lee L.M., Seftor E.A., Bonde G., Cornell R.A., Hendrix M.J. (2005). The fate of human malignant melanoma cells transplanted into zebrafish embryos: Assessment of migration and cell division in the absence of tumor formation. Dev. Dyn..

[4] Lu J.W., Ho Y.J., Yang Y.J., Liao H.A., Ciou S.C., Lin L.I., Ou D.L. (2015). Zebrafish as a disease model for studying human hepatocellular carcinoma. World J. Gastroenterol..

[5] Knox J.J., Cleary S.P., Dawson L.A. (2015). Localized and systemic approaches to treating hepatocellular carcinoma. J. Clin. Oncol..

[6] Venook A.P., Papandreou C., Furuse J., de Guevara L.L. (2010). The incidence and epidemiology of hepatocellular carcinoma: A global and regional perspective. Oncologist.

[7] Mueller S., Millonig G., Seitz H.K. (2009). Alcoholic liver disease and hepatitis C: A frequently underestimated combination. World J. Gastroenterol..

[8] Ferlay J., Steliarova-Foucher E., Lortet-Tieulent J., Rosso S., Coebergh J.W., Comber H., Forman D., Bray F. (2013). Cancer incidence and mortality patterns in Europe: Estimates for 40 countries in 2012. Eur. J. Cancer.

[9] Dhanasekaran R., Limaye A., Cabrera R. (2012). Hepatocellular carcinoma: Current trends in worldwide epidemiology, risk factors, diagnosis, and therapeutics. Hepat. Med..

[10] El-Serag H.B. (2012). Epidemiology of viral hepatitis and hepatocellular carcinoma. Gastroenterology.

[11] Llovet J.M., Burroughs A., Bruix J. (2003). Hepatocellular carcinoma. Lancet.

[12] Mallet V., Vallet-Pichard A., Pol S. (2011). The impact of human immunodeficiency virus on viral hepatitis. Liver Int..

[13] Schlachterman A., Craft W.W., Hilgenfeldt E., Mitra A., Cabrera R. (2015). Current and future treatments for hepatocellular carcinoma. World J. Gastroenterol..

[14] Cazejust J., Bessoud B., Colignon N., Garcia-Alba C., Planche O., Menu Y. (2014). Hepatocellular carcinoma vascularization: From the most common to the lesser known arteries. Diagn. Interv. Imaging.

[15] Yang Z.F., Poon R.T. (2008). Vascular changes in hepatocellular carcinoma. Anat. Rec..

[16] Dvorak H.F., Nagy J.A., Dvorak J.T., Dvorak A.M. (1988). Identification and characterization of the blood vessels of solid tumors that are leaky to circulating macromolecules. Am. J. Pathol..

[17] Bruix J., Sherman M. (2011). Management of hepatocellular carcinoma: An update. Hepatology.

[18] Ryder S.D. (2003). Guidelines for the diagnosis and treatment of hepatocellular carcinoma (HCC) in adults. Gut.

[19] Freeman R.B., Edwards E.B., Harper A.M. (2006). Waiting list removal rates among patients with chronic and malignant liver diseases. Am. J. Transplant..

[20] Lencioni R., Marrero J., Venook A., Ye S.L., Kudo M. (2010). Design and rationale for the non-interventional Global Investigation of Therapeutic DEcisions in Hepatocellular Carcinoma and Of its Treatment with Sorafenib (GIDEON) study. Int. J. Clin. Pract..

[21] Poon R.T., Fan S.T., Tsang F.H., Wong J. (2002). Locoregional therapies for hepatocellular carcinoma: A critical review from the surgeon’s perspective. Ann. Surg..

[22] Llovet J.M., Bruix J. (2003). Systematic review of randomized trials for unresectable hepatocellular carcinoma: Chemoembolization improves survival. Hepatology.

[23] Llovet J.M., Ricci S., Mazzaferro V., Hilgard P., Gane E., Blanc J.F., de Oliveira A.C., Santoro A., Raoul J.L., Forner A. (2008). Sorafenib in advanced hepatocellular carcinoma. N. Engl. J. Med..

[24] Gabrielson A., Tesfaye A.A., Marshall J.L., Pishvaian M.J., Smaglo B., Jha R., Dorsch-Vogel K., Wang H., He A.R. (2015). Phase II study of temozolomide and veliparib combination therapy for sorafenib-refractory advanced hepatocellular carcinoma. Cancer Chemother. Pharmacol..

[25] Verslype C., Van Cutsem E., Dicato M., Arber N., Berlin J.D., Cunningham D., De Gramont A., Diaz-Rubio E., Ducreux M., Gruenberger T. (2009). The management of hepatocellular cacinoma. Current expert opinion and recommendation derived from the 10th World Congress on Gastrointestinal Cancer, Barcelona, 2008. Ann. Oncol..

[26] Forner A., Llovet J.M., Bruix J. (2012). Hepatocellular carcinoma. Lancet.

[27] Kudo M. (2020). Recent Advances in Systemic Therapy for Hepatocellular Carcinoma in an Aging Society: 2020 Update. Liver Cancer.

[28] Engeszer R.E., Barbiano L.A., Ryan M.J., Parichy D.M. (2007). Timing and plasticity of shoaling behaviour in the zebrafish, Danio rerio. Anim. Behav..

[29] Streisinger G., Walker C., Dower N., Knauber D., Singer F. (1981). Production of clones of homozygous diploid zebra fish (Brachydanio rerio). Nature.

[30] Chen X., Li Y., Yao T., Jia R. (2021). Benefits of Zebrafish Xenograft Models in Cancer Research. Front. Cell Dev. Biol..

[31] Hason M., Bartunek P. (2019). Zebrafish Models of Cancer-New Insights on Modeling Human Cancer in a Non-Mammalian Vertebrate. Genes.

[32] White R.M., Sessa A., Burke C., Bowman T., LeBlanc J., Ceol C., Bourque C., Dovey M., Goessling W., Burns C.E. (2008). Transparent adult zebrafish as a tool for in vivo transplantation analysis. Cell Stem Cell.

[33] Lam S.H., Chua H.L., Gong Z., Lam T.J., Sin Y.M. (2004). Development and maturation of the immune system in zebrafish, Danio rerio: A gene expression profiling, in situ hybridization and immunological study. Dev. Comp. Immunol..

[34] Tang Q., Moore J.C., Ignatius M.S., Tenente I.M., Hayes M.N., Garcia E.G., Torres Y.N., Bourque C., He S., Blackburn J.S. (2016). Imaging tumour cell heterogeneity following cell transplantation into optically clear immune-deficient zebrafish. Nat. Commun..

[35] Goessling W., Sadler K.C. (2015). Zebrafish: An important tool for liver disease research. Gastroenterology.

[36] Delov V., Muth-Kohne E., Schafers C., Fenske M. (2014). Transgenic fluorescent zebrafish Tg(fli1:EGFP)y(1) for the identification of vasotoxicity within the zFET. Aquat. Toxicol..

[37] Tran T.C., Sneed B., Haider J., Blavo D., White A., Aiyejorun T., Baranowski T.C., Rubinstein A.L., Doan T.N., Dingledine R. (2007). Automated, quantitative screening assay for antiangiogenic compounds using transgenic zebrafish. Cancer Res..

[38] Barriuso J., Nagaraju R., Hurlstone A. (2015). Zebrafish: A new companion for translational research in oncology. Clin. Cancer Res..

[39] Liu J.S., Huo C.Y., Cao H.H., Fan C.L., Hu J.Y., Deng L.J., Lu Z.B., Yang H.Y., Yu L.Z., Mo Z.X. (2019). Aloperine induces apoptosis and G2/M cell cycle arrest in hepatocellular carcinoma cells through the PI3K/Akt signaling pathway. Phytomedicine.

[40] Chang W.T., Liu W., Chiu Y.H., Chen B.H., Chuang S.C., Chen Y.C., Hsu Y.T., Lu M.J., Chiou S.J., Chou C.K. (2017). A 4-Phenoxyphenol Derivative Exerts Inhibitory Effects on Human Hepatocellular Carcinoma Cells through Regulating Autophagy and Apoptosis Accompanied by Downregulating alpha-Tubulin Expression. Molecules.

[41] Wei P.L., Huang C.Y., Chang Y.J. (2019). Propyl gallate inhibits hepatocellular carcinoma cell growth through the induction of ROS and the activation of autophagy. PLoS ONE.

[42] Huang C.Y., Chang Y.J., Wei P.L., Hung C.S., Wang W. (2021). Methyl gallate, gallic acid-derived compound, inhibit cell proliferation through increasing ROS production and apoptosis in hepatocellular carcinoma cells. PLoS ONE.

[43] Xu J., Yan B., Zhang L., Zhou L., Zhang J., Yu W., Dong X., Yao L., Shan L. (2020). Theabrownin Induces Apoptosis and Tumor Inhibition of Hepatocellular Carcinoma Huh7 Cells Through ASK1-JNK-c-Jun Pathway. Onco. Targets. Ther..

[44] Tonon F., Zennaro C., Dapas B., Carraro M., Mariotti M., Grassi G. (2016). Rapid and cost-effective xenograft hepatocellular carcinoma model in Zebrafish for drug testing. Int. J. Pharm..

[45] Avci M.E., Keskus A.G., Targen S., Isilak M.E., Ozturk M., Atalay R.C., Adams M.M., Konu O. (2018). Development of a novel zebrafish xenograft model in ache mutants using liver cancer cell lines. Sci. Rep..

[46] Lin H.S., Huang Y.L., Wang Y.S., Hsiao E., Hsu T.A., Shiao H.Y., Jiaang W.T., Sampurna B.P., Lin K.H., Wu M.S. (2019). Identification of Novel Anti-Liver Cancer Small Molecules with Better Therapeutic Index than Sorafenib via Zebrafish Drug Screening Platform. Cancers.

[47] Yang J., Pei H., Luo H., Fu A., Yang H., Hu J., Zhao C., Chai L., Chen X., Shao X. (2017). Non-toxic dose of liposomal honokiol suppresses metastasis of hepatocellular carcinoma through destabilizing EGFR and inhibiting the downstream pathways. Oncotarget.

[48] Gunes A., Bagirsakci E., Iscan E., Cakan-Akdogan G., Aykutlu U., Senturk S., Ozhan G., Erdal E., Nart D., Barbet F.Y. (2018). Thioredoxin interacting protein promotes invasion in hepatocellular carcinoma. Oncotarget.

[49] Iscan E., Ekin U., Yildiz G., Oz O., Keles U., Suner A., Cakan-Akdogan G., Ozhan G., Nekulova M., Vojtesek B. (2021). TAp73beta Can Promote Hepatocellular Carcinoma Dedifferentiation. Cancers.

[50] Topel H., Bagirsakci E., Comez D., Bagci G., Cakan-Akdogan G., Atabey N. (2020). lncRNA HOTAIR overexpression induced downregulation of c-Met signaling promotes hybrid epithelial/mesenchymal phenotype in hepatocellular carcinoma cells. Cell Commun. Signal..

[51] van der Helm D., Groenewoud A., de Jonge-Muller E.S.M., Barnhoorn M.C., Schoonderwoerd M.J.A., Coenraad M.J., Hawinkels L.J.A.C., Snaar-Jagalska B.E., van Hoek B., Verspaget H.W. (2018). Mesenchymal stromal cells prevent progression of liver fibrosis in a novel zebrafish embryo model. Sci. Rep..

[52] Nakabayashi H., Taketa K., Miyano K., Yamane T., Sato J. (1982). Growth of human hepatoma cells lines with differentiated functions in chemically defined medium. Cancer Res..

[53] Zhao H., Desai V., Wang J., Epstein D.M., Miglarese M., Buck E. (2012). Epithelial-mesenchymal transition predicts sensitivity to the dual IGF-1R/IR inhibitor OSI-906 in hepatocellular carcinoma cell lines. Mol. Cancer Ther..

[54] Wang R.C., Huang C.Y., Pan T.L., Chen W.Y., Ho C.T., Liu T.Z., Chang Y.J. (2015). Proteomic Characterization of Annexin l (ANX1) and Heat Shock Protein 27 (HSP27) as Biomarkers for Invasive Hepatocellular Carcinoma Cells. PLoS ONE.

[55] Aden D.P., Fogel A., Plotkin S., Damjanov I., Knowles B.B. (1979). Controlled synthesis of HBsAg in a differentiated human liver carcinoma-derived cell line. Nature.

[56] Heffelfinger S.C., Hawkins H.H., Barrish J., Taylor L., Darlington G.J. (1992). SK HEP-1: A human cell line of endothelial origin. Vitr. Cell Dev. Biol..

[57] Fujise K., Nagamori S., Hasumura S., Homma S., Sujino H., Matsuura T., Shimizu K., Niiya M., Kameda H., Fujita K. (1990). Integration of hepatitis B virus DNA into cells of six established human hepatocellular carcinoma cell lines. Hepatogastroenterology.

[58] Nagamori S., Fujise K., Hasumura S., Homma S., Sujino H., Matsuura T., Shimizu K., Niiya M., Kameda H. (1988). Protein secretion of human cultured liver cells. Hum. Cell.

[59] Park J.G., Lee J.H., Kang M.S., Park K.J., Jeon Y.M., Lee H.J., Kwon H.S., Park H.S., Yeo K.S., Lee K.U. (1995). Characterization of cell lines established from human hepatocellular carcinoma. Int. J. Cancer.

[60] Anantharaju P.G., Gowda P.C., Vimalambike M.G., Madhunapantula S.V. (2016). An overview on the role of dietary phenolics for the treatment of cancers. Nutr. J..

[61] Jacobi H., Eicke B., Witte I. (1998). DNA strand break induction and enhanced cytotoxicity of propyl gallate in the presence of copper(II). Free Radic. Biol. Med..

[62] Rahman N., Jeon M., Kim Y.S. (2016). Methyl gallate, a potent antioxidant inhibits mouse and human adipocyte differentiation and oxidative stress in adipocytes through impairment of mitotic clonal expansion. Biofactors.

[63] Kim H., Lee G., Sohn S.H., Lee C., Kwak J.W., Bae H. (2016). Immunotherapy with methyl gallate, an inhibitor of Treg cell migration, enhances the anti-cancer effect of cisplatin therapy. Korean J. Physiol. Pharmacol..

[64] Wang Q., Gong J., Chisti Y., Sirisansaneeyakul S. (2016). Production of theabrownins using a crude fungal enzyme concentrate. J. Biotechnol..

[65] Nakachi K., Matsuyama S., Miyake S., Suganuma M., Imai K. (2000). Preventive effects of drinking green tea on cancer and cardiovascular disease: Epidemiological evidence for multiple targeting prevention. Biofactors.

[66] Baiz D., Pozzato G., Dapas B., Farra R., Scaggiante B., Grassi M., Uxa L., Giansante C., Zennaro C., Guarnieri G. (2009). Bortezomib arrests the proliferation of hepatocellular carcinoma cells HepG2 and JHH6 by differentially affecting E2F1, p21 and p27 levels. Biochimie.

[67] Baiz D., Dapas B., Farra R., Scaggiante B., Pozzato G., Zanconati F., Fiotti N., Consoloni L., Chiaretti S., Grassi G. (2014). Bortezomib effect on E2F and cyclin family members in human hepatocellular carcinoma cell lines. World J. Gastroenterol..

[68] Farra R., Dapas B., Baiz D., Tonon F., Chiaretti S., Del S.G., Rustighi A., Elvassore N., Pozzato G., Grassi M. (2015). Impairment of the Pin1/E2F1 axis in the anti-proliferative effect of bortezomib in hepatocellular carcinoma cells. Biochimie.

[69] Grassi G., Dawson P., Guarnieri G., Kandolf R., Grassi M. (2004). Therapeutic potential of hammerhead ribozymes in the treatment of hyper-proliferative diseases. Curr. Pharm. Biotechnol..

[70] Grassi G., Marini J.C. (1996). Ribozymes: Structure, function, and potential therapy for dominant genetic disorders. Ann. Med..

[71] Farra R., Dapas B., Pozzato G., Giansante C., Heidenreich O., Uxa L., Zennaro C., Guarnieri G., Grassi G. (2010). Serum response factor depletion affects the proliferation of the hepatocellular carcinoma cells HepG2 and JHH6. Biochimie.

[72] Farra R., Scaggiante B., Guerra C., Pozzato G., Grassi M., Zanconati F., Perrone F., Ferrari C., Trotta F., Grassi G. (2017). Dissecting the role of the elongation factor 1A isoforms in hepatocellular carcinoma cells by liposome-mediated delivery of siRNAs. Int. J. Pharm..

[73] Farra R., Dapas B., Pozzato G., Scaggiante B., Agostini F., Zennaro C., Grassi M., Rosso N., Giansante C., Fiotti N. (2011). Effects of E2F1-cyclin E1-E2 circuit down regulation in hepatocellular carcinoma cells. Dig. Liver Dis..

[74] Scaggiante B., Farra R., Dapas B., Baj G., Pozzato G., Grassi M., Zanconati F., Grassi G. (2016). Aptamer targeting of the elongation factor 1A impairs hepatocarcinoma cells viability and potentiates bortezomib and idarubicin effects. Int. J. Pharm..

[75] Grassi G., Schneider A., Engel S., Racchi G., Kandolf R., Kuhn A. (2005). Hammerhead ribozymes targeted against cyclin E and E2F1 cooperate to down-regulate coronary smooth muscle cell proliferation. J. Gene Med..

[76] Zhao Y., Wang X., Wang T., Hu X., Hui X., Yan M., Gao Q., Chen T., Li J., Yao M. (2011). Acetylcholinesterase, a key prognostic predictor for hepatocellular carcinoma, suppresses cell growth and induces chemosensitization. Hepatology.

[77] Eun J.R., Jung Y.J., Zhang Y., Zhang Y., Tschudy-Seney B., Ramsamooj R., Wan Y.J., Theise N.D., Zern M.A., Duan Y. (2014). Hepatoma SK Hep-1 cells exhibit characteristics of oncogenic mesenchymal stem cells with highly metastatic capacity. PLoS ONE.

[78] Hahm E.R., Singh S.V. (2007). Honokiol causes G0-G1 phase cell cycle arrest in human prostate cancer cells in association with suppression of retinoblastoma protein level/phosphorylation and inhibition of E2F1 transcriptional activity. Mol. Cancer Ther..

[79] Perrone F., Craparo E.F., Cemazar M., Kamensek U., Drago S.E., Dapas B., Scaggiante B., Zanconati F., Bonazza D., Grassi M. (2021). Targeted delivery of siRNAs against hepatocellular carcinoma-related genes by a galactosylated polyaspartamide copolymer. J. Control. Release.

[80] Dong D., Fu N., Yang P. (2016). MiR-17 Downregulation by High Glucose Stabilizes Thioredoxin-Interacting Protein and Removes Thioredoxin Inhibition on ASK1 Leading to Apoptosis. Toxicol. Sci..

[81] Kwon H.J., Won Y.S., Suh H.W., Jeon J.H., Shao Y., Yoon S.R., Chung J.W., Kim T.D., Kim H.M., Nam K.H. (2010). Vitamin D3 upregulated protein 1 suppresses TNF-alpha-induced NF-kappaB activation in hepatocarcinogenesis. J. Immunol..

[82] Melino G., De Laurenzi V., Vousden K.H. (2002). p73: Friend or foe in tumorigenesis. Nat. Rev. Cancer.

[83] Stiewe T., Putzer B.M. (2002). Role of p73 in malignancy: Tumor suppressor or oncogene?. Cell Death. Differ..

[84] Gupta R.A., Shah N., Wang K.C., Kim J., Horlings H.M., Wong D.J., Tsai M.C., Hung T., Argani P., Rinn J.L. (2010). Long non-coding RNA HOTAIR reprograms chromatin state to promote cancer metastasis. Nature.

[85] Deng J., Yang M., Jiang R., An N., Wang X., Liu B. (2017). Long Non-Coding RNA HOTAIR Regulates the Proliferation, Self-Renewal Capacity, Tumor Formation and Migration of the Cancer Stem-Like Cell (CSC) Subpopulation Enriched from Breast Cancer Cells. PLoS ONE.

[86] Lecharpentier A., Vielh P., Perez-Moreno P., Planchard D., Soria J.C., Farace F. (2011). Detection of circulating tumour cells with a hybrid (epithelial/mesenchymal) phenotype in patients with metastatic non-small cell lung cancer. Br. J. Cancer.

[87] Bouattour M., Raymond E., Qin S., Cheng A.L., Stammberger U., Locatelli G., Faivre S. (2018). Recent developments of c-Met as a therapeutic target in hepatocellular carcinoma. Hepatology.

[88] Marcellin P., Kutala B.K. (2018). Liver diseases: A major, neglected global public health problem requiring urgent actions and large-scale screening. Liver Int..

[89] Najimi M., Berardis S., El-Kehdy H., Rosseels V., Evraerts J., Lombard C., El Taghdouini A., Henriet P., van Grunsven L., Sokal E.M. (2017). Human liver mesenchymal stem/progenitor cells inhibit hepatic stellate cell activation: In vitro and in vivo evaluation. Stem Cell Res. Ther..

[90] Jang Y.O., Kim M.Y., Cho M.Y., Baik S.K., Cho Y.Z., Kwon S.O. (2014). Effect of bone marrow-derived mesenchymal stem cells on hepatic fibrosis in a thioacetamide-induced cirrhotic rat model. BMC Gastroenterol..

